# Manifesto: towards a clinically-oriented psychometrics

**DOI:** 10.1186/s12955-017-0655-3

**Published:** 2017-04-26

**Authors:** Andrew J. Vickers, Ling Y. Chen

**Affiliations:** Department of Epidemiology and Biostatistics, Memorial Sloan Kettering Cancer Center, 485 Lexington Avenue, New York, NY 10017 USA

## Abstract

**Background:**

New technologies to collect patient - reported outcomes have substantially solved the challenge of integrating a questionnaire in a busy clinical practice. At Memorial Sloan Kettering, we have been collecting patient reported outcomes electronically for many years. Our experience confirms the predicted benefits of obtaining patient reported outcomes but has also raised serious concerns about whether instruments developed for the research setting are appropriate for routine clinical use.

**Discussion:**

We summarize four principles for a clinically - relevant psychometrics. First, minimize patient burden: the use of a large number of items for a single domain may be of interest for research but additional items have little clinical utility. Secondly, use simplified language: patients who do not have good language skills are typically excluded from research studies but will nonetheless present in clinical practice. Third, avoid dumb questions: many questionnaire items are inappropriate when applied to a more general population. Fourth, what works for the group may not work for the individual: group level statistics used to validate survey instruments can obscure problems when applied to a subgroup of patients.

**Conclusion:**

There is a need for a clinically-oriented psychometrics to help design, test, and evaluate questionnaires that would be used in routine practice. Developing statistical methods to optimize questionnaires will be highly challenging but needed to bring the potential of patient reported outcomes into widespread clinical use.

It has long been argued that patient-reported outcomes based on validated instruments should be integrated into routine clinical care. The primary rationale is that the clinician-patient interaction often leads to underreporting of symptoms. This may result from clinicians not asking the right questions or not listening to patients’ answers, but there are also reasons to believe that patients sometimes feel inhibited discussing symptoms with doctors. Whatever the cause, it is surely not good medicine when we find that, for instance, the rate of appetite loss reported by cancer patients is six times higher than the rate documented in case notes [1], or where drug-related toxicities are reported at a 50% higher incidence by rheumatology patients than their doctors [2].

Yet use of patient-reported outcomes as a clinical routine has, for many years, been no more than an aspiration. In principle, giving patients a paper questionnaire, scoring it and then providing the results to the doctor, should improve the quality of the clinical consultation. In practice, administering paper questionnaires in a busy clinic and then fitting in scoring and reporting within clinic workflow requires a significant ongoing commitment of time and resources that is infeasible for many practices.

The development of technologies to collect patient-reported outcomes electronically has been transformative. Many institutions now email patients a link to an online questionnaire that is scored automatically and a report entered into the electronic medical record well in time for the doctor to review before the consultation [3].

At our institution, Memorial Sloan Kettering Cancer Center (MSKCC), we have used electronic methods to obtain patient-reported outcomes for many years. For instance, prostate cancer patients attending follow-up after surgery complete a questionnaire about urinary and erectile function [4] before their consultation. Recovery over time is then plotted on a graph that is included in the case notes that the surgeon reviews before seeing the patient (see Fig. 1). We have numerous other examples where patient-reported outcomes are part of routine care, including breast reconstruction; active surveillance for prostate cancer; chronic pain; recovery after hysterectomy; recovery after rectal cancer surgery; “red flag” symptoms in the immediate postoperative period; external beam radiotherapy for prostate cancer; “Rapid Fitness Assessment” for geriatric patients; high-risk breast clinic and neurosurgery. A number of other initiatives are in a pilot phase.

**Fig. 1:**
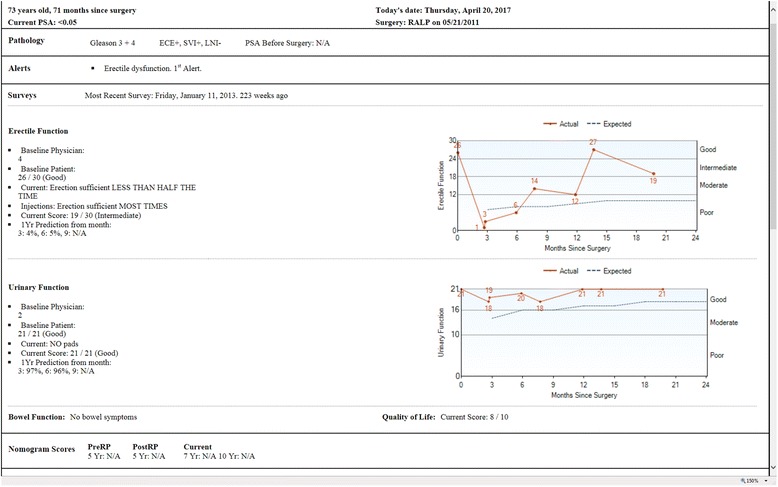
Example of a report seen by a clinician at Memorial Sloan Kettering Cancer Center during follow-up after surgery for prostate cancer

Our overall experience confirms the predicted benefits on incorporating patient-reported outcomes into clinical practice. Doctors tell us that it generally improves the consultation by allowing them to focus on the most relevant clinical issues. Take, for instance, the doctor of the patient shown in Fig. 1. Instead of starting the consultation with a series of questions to establish the patient’s status (“do you ever have to rush to the bathroom?”, “are you able to have sex?” etc.) the doctor asked the patient “your urinary function seems to be pretty good but you still seem to be having problems with erectile function. In fact, it seems to be getting worse. Is that right? Do you want to talk about that?”. Another common experience is when first introducing patient-reported outcomes in a clinic, when the doctor first receives the results of a questionnaire and says something like: “She never told me about that”. We have also found very high concordance between patient and doctor ratings of post-surgical symptoms.

On the other hand, our experience has raised serious questions about a different aspect of patient-reported outcomes, namely, how patient-reported outcomes instruments are developed. The methodology of questionnaire development has a long history and follows well-established principles. For instance, where several items are combined into a single domain score, the investigators designing the questionnaire assess the correlation between those items in order to determine whether they are indeed measuring the same underlying construct. Questionnaire developers will also want to see if domain scores are associated with known correlates, such as scores declining with age or improving after treatment.

We believe that such psychometric concerns are necessary, but far from sufficient, for designing a questionnaire that will be useful for routine clinical practice. Indeed, we would go further and say that because patient-reported outcome instruments have been almost exclusively designed for and used in the research setting, they often require a considerable redesign for clinical use. In this commentary, we summarize our clinical experience of using patient-reported outcomes in terms of four principles for a clinically-relevant psychometrics.

## Minimize patient burden

Selected patients volunteering for a research study might well be prepared to answer long and tedious questionnaires. This is certainly not the case for a typical patient just trying to get help for a medical problem. For instance, in one randomized trial [5], picked pretty much at random, patients completed the Patient Global Assessment of Arthritis, Patient Assessment of Arthritis Pain, Health Assessment Questionnaire-Disability Index (HAQ, 38 items), SF 36 (36 items), Functional Assessment of Chronic Illness Therapy-Fatigue (FACT-F, 35 items) and Medical Outcomes Study Sleep (MOS, Sleep 12 items). We have serious concerns as to the proportion of patients would be prepared to fill well over 100 items repeatedly, as part of routine practice. Moreover, we believe that it is completely unnecessary. From a clinical perspective, we do not see the value of asking about physical functioning on three different questionnaires (HAQ, SF36 and FACT-F), pain on three (two patient assessments plus SF36) or functional and emotional well-being on two (SF36 and FACT-F).

**Fig. 2:**
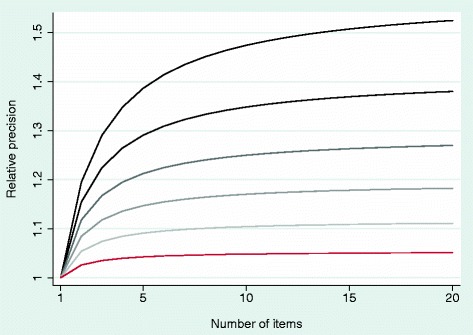
Relative precision of a domain score by number of items. *Shaded lines* vary from correlation between items of 0.40 (*light grey line*) to 0.90 (*black line*)

Moreover, we question the use of large numbers of items on a particular domain, such as 12 questions about sleep or 18 on fatigue, on purely statistical grounds. The purpose of including multiple items in a domain that is averaged into a single score is to reduce variance and improve precision. Figure 2 shows how precision increases as more items are included in a domain scale. Even if the correlation between items on a domain is relatively moderate, it is hard to justify including more than five or six items in a domain score. For instance, the items of “Functional Well-Being” domain of the FACT scale [6] have an average correlation close to 0.55. The relative increase in precision associated with including seven items compared to six is from 1.265 to 1.276, an improvement of 0.8%. In many cases, the results are even more extreme. For the BreastQ questionnaire [7], correlation between items on the “Satisfaction with Medical Team” domain is 0.84; for the Orgasm domain of the Female Sexual Function Index [8], the correlation is 0.93.

Accordingly, we try to keep the total number of items on a questionnaire to the minimum, certainly no more than 15 – 20. This is primarily achieved by reducing the number of items asked about a particular domain and by limiting the number of domains on a questionnaire.

## Use simplified language

Patients who do not have good language skills or use language that does not conform to that used by medical professionals are not typically invited to join research studies. For instance, it has been demonstrated that patients in underserved communities use different terms for intimate concerns such as urination, defecation and intercourse [9]. If a researcher invited a male patient to take part in a study on “bowel function” and the patient responded “what is that?”, the researcher would no doubt move on. Yet that patient would nonetheless present in clinic and we would still want him or her to provide information about symptoms. The items we use at MSKCC on clinical practice questionnaires therefore often include simplified language. For instance, when we ask about urination, we add “when you need to pee”; questions about bowel function are described as “when you sit down to go to the bathroom to pass solid waste”.

## Avoid dumb questions

The most widely used general quality-of-life questionnaire used in cancer research is the EORTC QLQ 30. The first five questions ask about activities of daily living, starting by asking whether patient has trouble doing strenuous activities and ending whether he or she needs help with self-care. This means that, for instance, a patient responding “not at all” to whether they have trouble taking a long walk would then be asked whether they have trouble taking a short walk. A comparable example comes from the International Index of Erectile Function (IIEF). A patient answering “I am not sexually active” in response to a question as to whether they find it difficult to get an erection would then nonetheless subsequently be asked whether they find it difficult to maintain their erection during sex. We have actually seen patients write “ok, ok, don’t make me feel bad” in the margin of a questionnaire.

Many questionnaire items can be inappropriate for subgroups of patients irrespective of their answers to other questions. A good example is the Expanded Prostate Cancer Index Composite (EPIC) questionnaire for prostate cancer patients. The instrument was developed in the traditional way, by gathering together a group of patients, asking about their symptoms and then designing items based on those symptoms. The problem is that symptoms in men with localized prostate cancer - the target group for the instrument - result from treatment, and there are a variety of different treatments for prostate cancer with different symptoms associated with each. Some prostate cancer patients experience tender breasts and hot flashes from hormonal therapy and others have bowel symptoms as a side-effect of radiation. But asking a surgery patient whether they have a problem with “losing control of [their] stools” or whether they have blood in their stools makes no sense at all. A patient on conservative management asked whether he has been having hot flashes or breast enlargement is likely to be similarly baffled. We have actually seen patients annotate paper questionnaires with comments such as “I’m not a woman you know”.

We take three approaches to avoid the “dumb question” problem. First, we use different instruments for different groups of patients depending on their expected symptoms. At a cancer hospital, this generally means stratifying patients by treatment. Second, we read each question extremely carefully to determine whether it is appropriate for all of the patients we would see in routine practice. It sounds like a simplistic and obvious approach, but we have been struck by the number of times that clinicians recommend questionnaires without a thorough knowledge of individual items. Third, we use skip and branch logic. For instance, a patient reporting that they are not sexually active in response to a first question about erectile function is not asked further questions about sexual activity.

## What works for the group may not work for the individual

Survey instruments are validated by providing group level statistics, such as correlations. This can obscure important problems with a questionnaire when applied to a subgroup of patients. As an example, the FSFI includes a domain on pain during sex. Items such as “rate your level … of pain during or following vaginal penetration” are scored from 5 for “very low or none” to 1 for “very high”. Importantly, if the respondent says that she “did not attempt intercourse”, she is scored as a 0, more symptomatic than very high pain. At a group level this works reasonable well, because penetration is part of sex for most sexually active women. But women may not engage in penetrative sex for a whole host of reasons, and it may well be inaccurate to rate these women as having extremely high levels of pain. There is a directly analogous problem for the IIEF, the instrument for male sexual function. Several questions refer to penetration and other ask about intercourse, with the response “I did not attempt intercourse in the past month” receiving the lowest score. It should be obvious that men may not have had recent intercourse for reasons other than erectile dysfunction, the most obvious being that they are not currently in a heterosexual relationship.

Our approach at MSKCC is to take a critical look at each and every item on a questionnaire and think through what patient factors might lead to misleading responses. In the case of the intercourse question, for example, we have added a question for men responding no recent intercourse in terms of the reason for lack of intercourse, in brief, lack of a willing or available partner vs. lack of confidence or ability to have an erection.

## Conclusions

It should not be surprising that instruments designed for research studies are often inappropriate for clinical use. For instance, the IIEF was originally developed for a study that specified in the eligibility criteria that participants should be men attempting penetrative sex with a long-term female partner [10]. Such a study would not indicate whether IIEF is appropriate for single men, gay men or those who have sex without intercourse, all of whom present in clinical practice. Table 1 highlights the issues that we have found using research instruments in routine clinical care.

**Table 1:** Comparison between what is known about use of PRO instruments for routine clinical practice and new issues highlighted in this commentary

What is known	What is highlighted
PROs instruments have generally been developed for research purposes. They tend to be long in order to maximize the amount of data available for researchers to analyze.	Long research questionnaires are not practical as part of routine care. Patients who have not specifically volunteered to complete questionnaires may have poor compliance with time-consuming instruments. No more than 15–20 items are recommended in a questionnaire,with no more than 5 – 7 items in a domain.
Patient who do not have good language skills are not typically invited on research studies.	Patients with low language skills present in clinics. Questionnaires need to include simplified language.
Instruments are traditionally developed by gathering a group of patients, asking about their symptoms and designing items based on wide cross-section of symptoms.	Many questionnaire items can be inappropriate for specific subgroups of patients. Different instruments sometimes need to be used for different groups of patients, depending on their expected symptoms.
Survey instruments are validated by providing group level statistics.	Group level statistics can obscure problems when applying an instrument to certain subgroups. It is important to critically look at each item and think through what might lead to misleading responses.
Research design and statistical methods for psychometric studies has focused on instruments for research use.	New designs and methods are needed to develop instruments for clinical use.

We call for a clinically-oriented psychometrics to help design, test and evaluate questionnaires that would be feasible to use in routine practice and that would provide useful information to clinicians to help them counsel and treat patients. In our experience, the key to developing such questionnaires has been to watch real patients completing them in routine practice, and then to debrief those patients about their experience. It is remarkable the degree to which patients interpret survey questions for their individual circumstances in a way that researchers and clinicians do not expect. As yet, however, our methodology has been informal and non-quantitative and it is difficult to see how we might quantify small changes to a questionnaire, such as clarification of a word or concept, that affect only a few patients. We predict that developing statistical methods to optimize questionnaires for clinical use will be highly challenging. But such methods will undoubtedly be needed if we are to bring the potential of patient-reported outcomes into widespread clinical use.
