# Erratum to: Manifesto: towards a clinically-oriented psychometrics

**DOI:** 10.1186/s12955-017-0684-y

**Published:** 2017-05-22

**Authors:** Andrew J. Vickers, Ling Y. Chen

**Affiliations:** Department of Epidemiology and Biostatistics, Memorial Sloan Kettering Cancer Center, 485 Lexington Avenue, New York, NY 10017 USA

## Erratum

The original article [1] contains an error whereby the caption of Fig. 2 incorrectly describes respective lines in the associated graph.

**Fig. 2:**
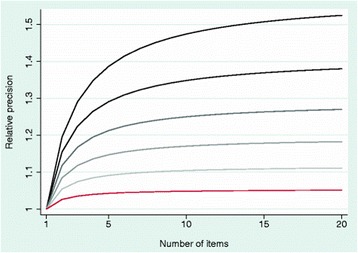
Relative precision of a domain score by number of items. ***Shaded lines*** vary from correlation between items of 0.40 (***black line***) to 0.90 (***red line***)

The article has now been updated so that the caption to Fig. 2 correctly reads as such:

Relative precision of a domain score by number of items. *Shaded lines* vary from correlation between items of 0.40 (*black line*) to 0.90 (*red line*)
